# Molecular epidemiology of paediatric invasive pneumococcal disease in Andalusia, Spain

**DOI:** 10.1017/S0950268822001376

**Published:** 2022-08-22

**Authors:** Beatriz de Felipe, Pablo Obando Pacheco, Begoña Carazo Gallego, David López Martín, Juan Luis Santos Pérez, Yolanda González Jiménez, María José Muñoz Vilches, Nerea Cardelo Autero, Verónica González Galán, Francisco José Morón, Juan Antonio Cordero Varela, María José Torres Sánchez, Antonio Medina Claros, David Moreno Pérez, Ignacio Obando

**Affiliations:** 1 Biomedical Institute of Seville (IBiS), University of Seville, University Hospital Virgen del Rocío (HUVR)/CSIC, Seville, Spain; 2 Pediatric Unit, Hospital Universitario Virgen de Valme, Seville, Spain; 3 Pediatric Infectious Diseases and Immunodeficiencies Unit, Hospital Universitario Regional de Málaga, Malaga, Spain; 4 Pediatric Unit, Hospital Costa del Sol, Marbella, Málaga, Spain; 5 Pediatric Infectious Diseases and Immunodeficiencies Unit, Hospital Virgen de las Nieves, Granada, Spain; 6 Pediatric Pneumology Unit, Hospital Universitario San Cecilio, Granada, Spain; 7 Pediatric Infectious Diseases and Immunodeficiencies Unit, HUVR, Seville, Spain; 8 Microbiology Department, HUVR, IBiS, Seville, Spain; 9 Coordinador del Plan Andaluz de Vacunas de Andalucía (PVA), Pediatric Infectious Diseases and Immunodeficiencies Unit, Hospital Universitario Regional de Málaga, Malaga, Spain

**Keywords:** Genotypes, invasive pneumococcal disease, pneumococcal conjugate vaccine, serotypes, *Streptococcus pneumoniae*

## Abstract

This study aimed to assess the impact of the introduction of pneumococcal conjugate vaccine 13 (PCV13) on the molecular epidemiology of invasive pneumococcal disease (IPD) in children from Andalusia. A population-based prospective surveillance study was conducted on IPD in children aged <14 years from Andalusia (2018–2020). Pneumococcal invasive isolates collected between 2006 and 2009 in the two largest tertiary hospitals in Andalusia were used as pre-PCV13 controls for comparison of serotype/genotype distribution. Overall IPD incidence rate was 3.55 cases per 100 000 in 2018; increased non-significantly to 4.20 cases per 100 000 in 2019 and declined in 2020 to 1.69 cases per 100 000 (incidence rate ratio 2020 *vs.* 2019: 0.40, 95% confidence interval (CI) 0.20–0.89, *P* = 0.01). Proportion of IPD cases due to PCV13 serotypes in 2018–2020 was 28% (*P* = 0.0001 for comparison with 2006–2009). Serotypes 24F (15%) and 11A (8.3%) were the most frequently identified non-PCV13 serotypes (NVT) in 2018–2020. Penicillin- and/or ampicillin-resistant clones mostly belonged to clonal complex 156 (serotype 14-ST156 and ST2944 and serotype 11A-ST6521). The proportion of IPD cases caused by PCV13 serotypes declined significantly after the initiation of the PCV13 vaccination programme in 2016. Certain NVT, such as serotypes 24F and 11A, warrant future monitoring in IPD owing to invasive potential and/or antibiotic resistance rates.

## Introduction

*Streptococcus pneumoniae* is a commensal bacterium of the human respiratory tract. Under appropriate conditions, they become pathogenic and can cause high morbidity and mortality [1, 2]. This microorganism is a leading cause of mucosal diseases, such as otitis media, sinusitis and pneumonia. In addition, it is a prominent pathogen in invasive diseases, including bacteraemia, meningitis and sepsis.

Routine vaccination of infants and young children with pneumococcal conjugate vaccines (PCVs) has led to a prompt and sustained reduction in the incidence of invasive pneumococcal disease (IPD) rates. This reduction also extends to unvaccinated cohorts of older children and adults due to herd effect [3, 4]. Despite their success, PCVs have limitations: coverage is serotype dependent, and there is a potential increase in IPD caused by non-vaccine types (NVT) due to serotype replacement in their nasopharyngeal (NP) ecological niche [5].

Active surveillance in the epidemiology of IPD after PCVs introduction in different epidemiological settings is crucial to fully understand the effect of these vaccines on the incidence of IPD, the distribution of serotypes and whether there is some degree of evasion of vaccine immunity due to the increase in NVT in the IPD. Although epidemiological studies have suggested that capsular type is the primary determinant of invasiveness, a non-capsular genomic background may also influence the ability of pneumococci to cause invasive disease [6]. Therefore, to assess the impact of PCV introduction in the community, genotyping invasive isolates would provide additional and more complete information to classical studies based only on information regarding serotype distribution. This comprehensive approach would allow the identification of emerging serotypes/clones that are not included in current PCVs and knowledge of their invasive potential and resistance rates.

Analysis of the impact of PCVs on the incidence of IPD in Spain has been difficult because variations in vaccination policies, evaluation periods and surveillance systems could account for differences in reported data between geographical areas [7, 8]. Andalusia is the most populous region of Spain. Although PCV7 was introduced in 2001, vaccination coverage was partial because PCVs were only available in the private market until the introduction of PCV13 in the paediatric immunisation programme in December 2016. In this study, we aimed to characterise the epidemiology and circulating serotypes/genotypes in IPD and carriage in the paediatric population of selected provinces of Andalusia to gain an in-depth understanding of the impact of PCV13 introduction in Andalusia at the population level.

## Patients and methods

### Study setting and design

This was a population-based prospective active clinical and molecular surveillance study of laboratory-confirmed cases of IPD in children aged <14 years from January 2018 to December 2020. PCV13 uptake ranged from 97.2% in 2018 to 98.2% in 2020.

The study was conducted in 28 hospitals with paediatric departments located in three of the four most populated Andalusian provinces: Seville, Malaga and Granada. The total population <14 years under surveillance on 1 January 2018 was 684 694 (with 83 587, 114 181 and 486 926 persons aged <2, 2–4 and 5–13 years, respectively) according to the public data from the Statistical National Institute. IPD cases were defined as *S. pneumoniae* cultured from normal sterile fluid samples. The study protocol was approved by the local research ethics committee.

### Data collection

Basic demographic data (age, sex and PCV vaccination status), clinical presentation (meningitis, pneumonia or primary bacteraemia), underlying comorbidities and outcomes were retrieved from standardised case report forms completed by the attending physicians who managed the cases. The collected information was added anonymously to the dataset specifically designed for this study.

### Microbiological studies, serotyping and genotyping

All pneumococcal invasive isolates were cultured and identified in different participating health centres using standard laboratory protocols. All presumptive pneumococcal strains were sent to the Microbiology Department at the University Hospital Virgen del Rocio (HUVR) for antimicrobial susceptibility, serotyping and genotyping studies. Genomic DNA from *S. pneumoniae* isolates was fully sequenced, and serotypes were deduced bioinformatically based on the CPS locus [9]. In addition, some participating centres sent pneumococcal isolates to the Spanish National Reference Laboratory to complete serotype detection by the Quellung reaction, as they had been routinely performed prior to the study period. The PCV13 group (VT) included the serotypes included in the PCV13 vaccine. All other serotypes were considered non-PCV13 serotypes (NVT).

Genotyping was performed using multilocus sequence typing (MLST) as reported elsewhere [10]. A clonal complex (CC) was defined as sequence type (ST) sharing five out of seven allelic variants.

The susceptibility to antimicrobials was tested using an *e* test. interpretative minimum inhibitory concentration (MIC) breakpoints for antibiotics were defined according to the recommendations of the European Committee on Antimicrobial Susceptibility Testing. Meningitis breakpoints for *β*-lactam antibiotics were used in meningitis cases and non-meningitis breakpoints in all other cases. Intermediate and fully resistant strains were combined into the non-susceptible category.

### Pre-PCV13 historical controls

To compare the serotype distribution and antibiotic resistance rates between post-PCV13 and pre-PCV13, we used a historical collection of 75 isolates collected from paediatric patients with IPD in two major Andalusian tertiary paediatric hospitals (HUVR, Seville and Hospital Regional Universitario, Malaga) during the 2006–2009 period, partially included in a previous study [11].

### Invasive disease potential

The invasive potential of the serotypes identified in IPD cases was estimated by the odds ratio (OR) in relation to their prevalence in IPD and NP carriage. Invasive OR was calculated by referring to all other serotypes as follows: OR = (*ad*)/(*bc*), where *a* is the number of invasive A serotypes, *b* is the number of carriage A serotypes, *c* is the number of invasive non-A serotypes and *d* is the number of carriage non-A serotypes [12]. Serotypes with OR > 1 were considered to have an increased possibility of IPD. OR was calculated by adding a single carrier for the specific serotypes identified in IPD, but not in NP carriage.

A total of 76 colonising isolates were recovered from an NP carriage study conducted during the same period (2018–2020) in 394 healthy children aged 6 months to 5 years attending primary healthcare centres in Seville during a well-being visit. NP sampling was performed as previously reported, and written informed consent was obtained from the parent/guardian of the study children [13]. Pneumococci identification, serotyping and genotyping were performed at HUVR, as previously described.

### Statistical analyses

Statistical analyses were performed using SSPS 26 (SSPS Inc., Chicago, IL, USA) and Epidat 4.2 (Conselleria de Sanidade, Xunta de Galicia, Spain). Incidence rates (IRs) were calculated as the number of cases per 100 000 inhabitants, using population data from the Spanish census population estimates for the corresponding year. Incidence rate ratios (IRRs), with their respective 95% confidence intervals (CIs), were calculated by comparing incidence in different years. Categorical variables were analysed using the *χ*^2^ test and Fisher's exact test, as appropriate.

## Results

### Evolution of the IPD

A total of 63 IPD cases were identified during the study period. Thirty-one (49%) cases were diagnosed in children <2 years old, 21 (33%) cases in children 2–4 years old and 11 (17%) cases in children 5–13 years old. Pneumonia (with or without associated pleural empyema) was the most frequent clinical condition (*n* = 28, 44%), followed by primary bacteraemia (*n* = 22, 35%), meningitis (*n* = 12, 19%) and primary peritonitis (*n* = 1, 2%), respectively. A total of 40 (64%) and 4 (6%) patients had been immunised with PCV13 and PCV7, respectively. Nine (14%) children had pre-existing risk factors for IPD, including preterm, congenital heart disease and chronic pulmonary disease (*n* = 2 each) and haematopoietic stem cell transplantation, congenital CMV infection and primary immunodeficiency (*n* = 1 each). There were no deaths, and two (3%) patients with meningitis developed neurological sequelae.

**Fig. 1.**
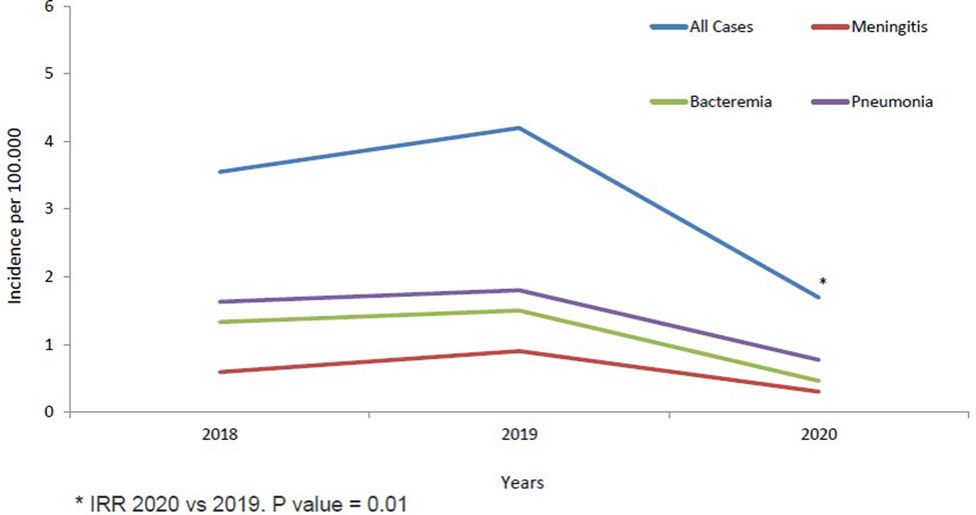
IPD incidence in children <14 years from 2018 to 2020 stratified by clinical conditions.

**Fig. 2.**
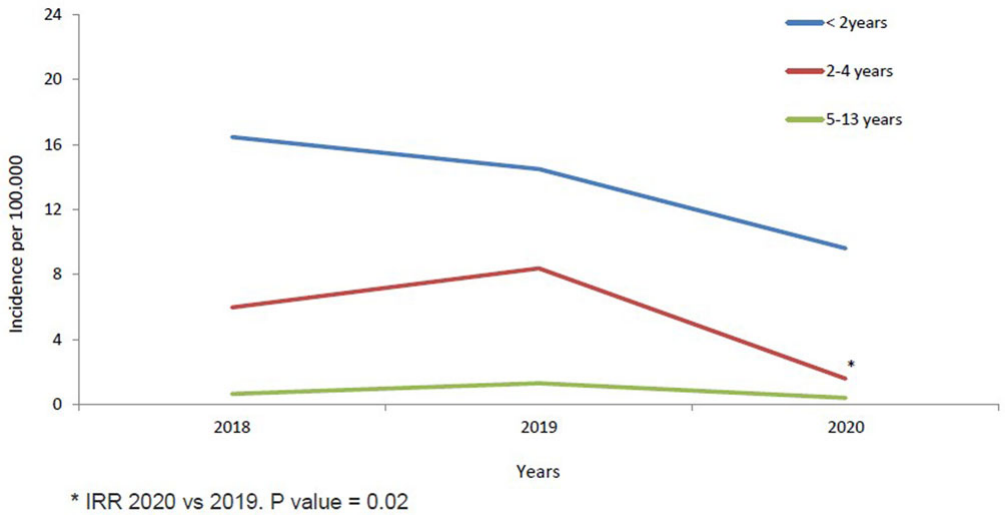
IPD incidence in children <14 years from 2018 to 2020 stratified by age groups.

The annual IR of IPD in children <14 years was 3.55/100 000 cases in 2018, increased non-significantly in 2019 to 4.20/100 000 cases (2019 *vs.* 2018; IRR: 1.18, 95% CI 0.69–2.04). IR in 2020 decreased significantly by 60% in 2020 to 1.69/100 000 cases, compared to 2019 (2020 *vs.* 2019; IRR: 0.40, 95% CI 0.20–0.81, *P* = 0.01) (Table 1 and Fig. 1). The temporal evolution of annual IRs stratified by clinical conditions and age groups are shown in Table 1 and Figures 1 and 2.

**Table 1.** IR of IPD and IRR in children <14 years globally and stratified by age groups and clinical conditions

Age group clinical condition	2018 incidence (per 100 000)	2019 incidence (per 100 000)	IRR 2019 *vs*. 2018 (95% CI)	2020 incidence (per 100 000)	IRR 2019 *vs*. 2018 (95% CI)
<14 years					
Global	3.55	4.20	1.18 (0.69–2.04)	1.69	0.40 (0.20–0.81)*
Pneumonia	1.63	1.80	1.11 (0.49–2.51)	0.77	0.43 (0.15–1.21)
Primary bacteraemia	1.33	1.50	1.13 (0.47–2.77)	0.46	0.41 (0.13–1.30)
Meningitis	0.59	0.90	1.52 (0.36–7.33)	0.30	0.34 (0.07–1.69)
<2 years					
Global	16.46	14.49	0.88 (0.39–1.96)	9.61	0.66 (0.26–1.71)
Pneumonia	6.33	5.27	0.83 (0.17–3.87)	4.12	0.78 (0.11–4.62)
Primary bacteraemia	7.60	5.27	0.69 (0.20–2.46)	4.12	0.78 (0.11–4.62)
Meningitis	2.53	3.95	1.56 (0.18–18.68)	1.37	0.35 (0.07–4.33)
2–4 years					
Global	5.97	8.38	1.39 (0.56–3.47)	1.59	0.19 (0.04–0.84)^†^
Pneumonia	2.23	3.05	1.36 (0.23–9.31)	1.59	0.52 (0.05–3.64)
Primary bacteraemia	2.23	3.81	1.70 (0.33–10.98)	0	–
Meningitis	1.49	1.52	1.02 (0.07–14.11)	0	
5–13 years					
Global	0.65	1.31	2.03 (0.43–12.53)	0.44	0.35 (0.07–1.71)
Pneumonia	0.65	0.87	1.34 (0.23–9.17)	0	–
Primary bacteraemia	0	0.22		0	–
Meningitis	0	0.22		0.22	1.01 (0.01–79.62)

IRR, incidence rate ratio.

*P value = 0.01, ^†^*P* value = 0.02.

### Serotype distribution

Twenty serotypes were identified among the 60 isolates available for serotyping/genotyping during the post-PCV13 period. Five serotypes were VT, and the remaining 15 serotypes were NVT. VT cases included four cases of vaccine failure in children who had been fully immunised with PCV13: pleural empyema and/or bacteraemic pneumonia due to serotypes 3 (*n* = 3) and 14 (*n* = 1), respectively. Additionally, two patients with a single dose of PCV13, aged 2- and 3-month-old patients, developed IPD due to serotypes 18C and 19A. The proportion of VT significantly decreased in 2018–2020 compared to historical controls in the pre-PCV13 period (28% *vs.* 88%; *P* = 0.0001).

NVT most frequently identified during the 2018–2020 period were according to their rank in IPD: 24F (*n* = 9, 15%), 11A (*n* = 5, 8.3%), 22F (*n* = 4, 6.7%), 8, 12F and 33F (*n* = 3, 5% each). The proportion of the first three NVT increased between the pre- and post-PCV13 periods (*P* = 0.01 for serotype 24F and *P* = 0.02 and 0.04 for serotypes 11A and 22F, respectively) (Fig. 1). On the other hand, the most prevalent VT in the contemporary period were: 3 (*n* = 6, 10%), 1 (*n* = 4, 6.7%) and 14 and 19A (*n* = 3, 5% each), with a significant decrease in the proportion of serotype 1 isolates in relation to the historical period (*P* = 0.0002). Finally, serotypes 5 and 7F, which circulated significantly in the pre-CNV13 period, were not identified during 2018–2020 (*P* = 0.03, 2018–2020 *vs.* 2006–2009).

### Genotype distribution

**Fig. 3.**
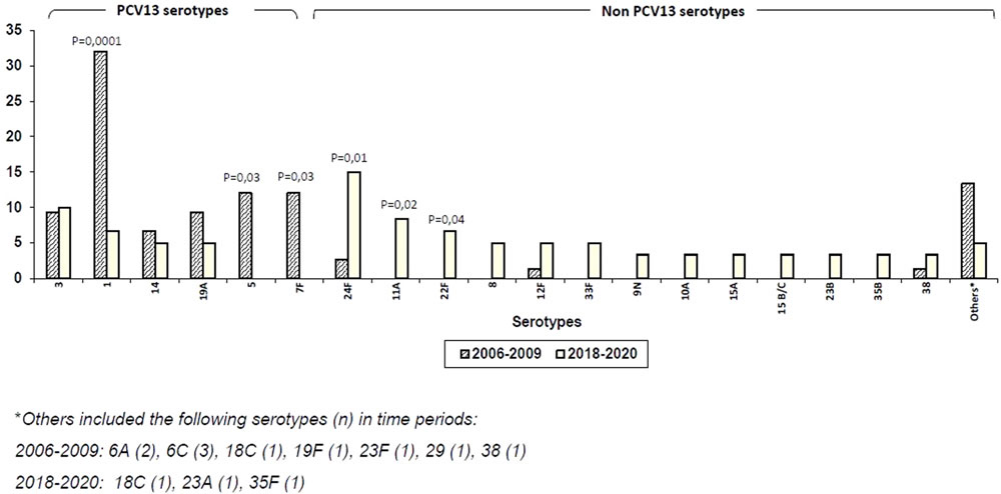
Distribution of serotypes causing IPD in the pre-PCV13 period (2006–2009) and post-PCV13 period (2018–2020).

Twenty-six different STs were identified by molecular typing using MLST in the post-PCV13 period, being CC230 (*n* = 9, 15%) and CC156 (*n* = 8, 13.3%) the most common clones in the post-PCV13 (Table 2). CC230, expressed as serotype 24F, was associated with pneumonia, primary bacteraemia and meningitis and comprised of two clones, ST230 and the single-locus variant (SLV) ST4677. ST230 circulated during both study periods, and ST4667 was only detected in the post-PCV13 period (Fig. 3).

**Table 2.** CCs and STs with ≥2 isolates for the study periods 2018–2020 or 2006–2009 and their associated serotypes

CC/ST	Serotype	2018–2020	2006–2009
		No. of cases (%)	No. of cases (%)
		*N* = 60	*N* = 75
CC230		9 (15)	2 (2.7)
ST230	24F	4 (6.7)	2 (2.7)
**ST4677**	24F	5 (8.3)	0
CC156		8 (13.3)	4 (5.3)
ST156	14	2 (3.3)	4 (5.3)
**ST2944**	14	1 (1.7)	0
**ST6521**	11A	5 (8.3)	0
ST180	3	5 (8.3)	4 (5.3)
ST306	1	4 (6.7)	15 (20)
**ST433**	22F	4 (6.7)	0
**ST1110**	8	3 (5)	0
**ST2372**		3 (5)	0
	19A	1 (1.7)	0
	23B	2 (3.3)	0
**ST717**	33F	3 (5)	0
ST260	3	1 (1.7)	3 (4)
ST393	38	2 (3.3)	1 (1.3)
**ST66**	9N	2 (3.3)	0
**ST1551**	10A	2 (3.3)	0
**ST1078**	15A	2 (3.3)	0
**ST1262**	15B/C	2 (3.3)	0
**ST2690**	35B	2 (3.3)	0
ST392	19A	1 (1.7)	2 (2.7)
ST1201	19A	1 (1.7)	2 (2.7)
ST228	1	0	9 (12)
CC289			9 (12)
ST289	5	0	3 (4)
ST1221	5	0	6 (8)
ST191	7F	0	9 (12)
ST1876	6C	0	2 (2.7)
ST276	19A	0	2 (2.7)

ST in bold were detected only during the 2018–2020 period.

CC156 was associated with serotypes 14 (ST156 and SLV ST2944) and 11A (double locus variant ST6521) in the 2018–2020 period and with serotypes 14 (ST156) and 9V (SLV ST838) in the 2006–2009 period. CC156 was associated with pneumonia and bacteraemia in serotype 14, and exclusively with primary bacteraemia in serotype 11A.

A significant decline was observed in the proportion of serotype 1 ST306 that was the most common clone in the pre-PCV13 period (from 20% to 6.3%, *P* = 0.04). Other highly prevalent clones in this period, such as serotype 5 CC289, serotype 7F ST191 and serotype 1 ST228, disappeared completely in the post-PCV13 period.

### Invasive potential

**Fig. 4.**
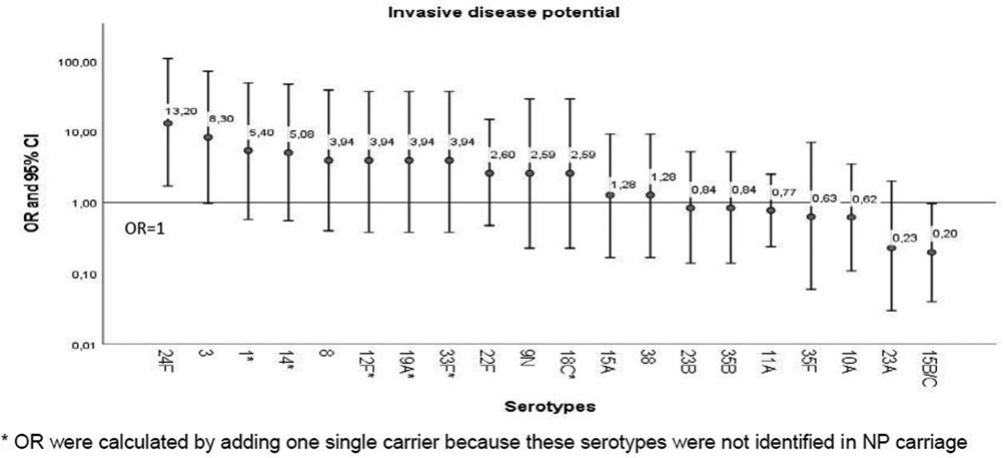
Invasive disease potential (ORs and 95% CIs) of the specific serotypes identified in IPD over the period 2018 until 2020. OR displayed on a logarithmic scale, error bars indicate 95% CIs.

There were important differences in the ORs of the different serotypes identified in the IPD (Fig. 4). Serotype 24F had significantly high invasive potential (OR 13.2, 95% 1.7–107.7), and serotype 3 was close to significance (OR 8.3, 95% CI 0.97–71.2). Serotypes 1, 14, 19A and 18C (VT) and serotypes 12F and 33F (NVT) had non-statistically significant highly invasive disease; however, ORs were calculated by adding a single carrier because these serotypes were not identified in NP carriage. The remaining serotypes had either non-statistically significant high invasive potential (serotypes 8, 22F, 9N, 15A and 38) or low invasive potential (serotypes 23B, 35B, 11A, 35F, 10A, 23A and 15B/C).

### Antimicrobial non-susceptibility

A total of 18 (28.6%) strains were penicillin non-susceptible (PNS) and five (7.9%) strains were penicillin-resistant (PR). Non-susceptibility and resistance rates to other *β*-lactam antibiotics commonly used in the treatment of IPD were as follows: ampicillin (12.7% and 12.7%), cefotaxime (7.9% and 0%) and ceftriaxone (3.2% and 0%). Fifteen (23.8%) strains were also erythromycin-resistant (ER).

Table 3 shows the distribution of the serotypes with reduced susceptibility to *β*-lactam antibiotics and erythromycin. Resistance to penicillin and/or ampicillin was detected mostly in strains belonging to CC156 (serotypes 11A and 14). Furthermore, there were two cases of meningitis due to serotype 24F that with meningitis breakpoint were also PR. This serotype was universally PNS (MIC 0.5–1 mg/l) and ER.

**Table 3.** Distribution of serotypes associated with reduced susceptibility to *β*-lactams antibiotics and erythromycin

Serotype	Proportion (%) of strains with reduced susceptibility (% resistant) to				
	PEN	AMP	CTX	CRO	ERY
11A	100 (0)	100 (100)	40 (0)	0 (0)	0
14	100 (100)	100 (100)	100 (0)	66.7 (0)	0
15A	0	0	0	0	100 (100)
23A	0	0	0	0	100 (100)
23B	100 (0)	0	0	0	0
24F	100 (22.2^a^)	0	0	0	100 (100)
33F	0	0	0	0	100 (100)
Total	28.6 (7.9)	12.7 (12.7)	7.9 (0)	3.2 (0)	23.8 (23.8)

PEN, penicillin; AMP, ampicillin; CTX, cefotaxime; CRO, ceftriaxone; ERY, erythromycin.

a Meningitis breakpoint.

## Discussion

The present study is the first report of the evolution of IPD among Andalusian children after the introduction of the PCV13 universal childhood vaccination, and it was conducted in parallel with an NP carriage study and clonal analysis of the invasive isolates. The annual IR of IPD in <14 years peaked in 2019 at 4.20/100 000 cases and decreased by 60% in 2020. The temporal evolution of the IR during the study period is difficult to interpret because of the disruptive effects of the COVID-19 pandemic. In this regard, in a comprehensive study with data from 26 countries and territories, the incidence of reported *S. pneumoniae* infections decreased by 68% at 4 weeks and 82% at 8 weeks following the week when significant changes in population movements were recorded [14]. Furthermore, marked reductions in bacteraemic pneumococcal pneumonia and non-pneumonia IPD were reported in Israel during the COVID-19 pandemic (IRR January 2020 through February 2021 *vs.* mean monthly rates of 0.19 and 0.42, respectively, in 2016–2019) [15]. The decline in pneumococcus-associated disease was temporally associated with the full suppression of respiratory syncytial virus (RSV), influenza virus and human metapneumovirus (hMPV) circulation. To better understand the impact of the COVID-19 pandemic, PCV13 and viral circulation on IPD epidemiology, it is advisable to continue monitoring the evolution of IPD in the coming years.

The proportion of IPD cases due to VT decreased by 68% between 2018–2020 and 2006–2009 and this was due to the eradication of serotypes 5 and 7F, which were widely represented in the pre-VCN13 period. In addition, there was a marked reduction (83%) in the circulation of serotype 1, the most prevalent serotype in the historical period [7]. Similar findings were reported in Madrid in a Heracles study, except for a more pronounced decline in serotype 19A IPD rates. This was the most frequently detected serotype in Madrid during the pre-PCV13 period, related to the expansion of the multi-resistant clone ST320, a phenomenon not detected in our geographical area. On the other hand, serotype 3, the most common VT identified in the study period, caused three of the four breakthrough IPD in children fully vaccinated with PCV13. Serotype 3 is currently one of the leading causes of breakthrough IPD in paediatric patients, which might likely reflect the reduced vaccine effectiveness of PCV13 against serotype 3 compared to other serotypes [16–18]. Serotype 3 declined by 32% between 2009 and 2019 in Spanish children. Nevertheless, it remains an important cause of paediatric IPD, as in certain European countries [19–21].

Sixteen NVT cases were identified, with 24A, 11A and 22F being the most prevalent. They showed differences in invasive potential, antibiotic resistance and coverage by the new generation of PCV vaccines licensed by the Food and Drug Administration for adults aged ≥18 years (15-valent PCV (PCV15) and 20-valent PCV (PCV20)). According to the distribution of serotypes in 2018–2020, the estimated coverage would have been 41.7% for PCV15 and 68.8% for PCV20, respectively.

The wide distribution of NVT in IPD is a predictable finding previously reported in a systematic review and meta-analysis based on epidemiological surveillance data for IPD in 20 countries where PCV10/VCN13 had been introduced [22]. In this review, the prevalence of NVT (22F, 12F, 33F, 24F, 15C, 15 B, 23 B, 10A, 38 and 15A) did not differ significantly from those identified in the present study. These serotypes, as well as other emerging serotypes in recent years, such as serotypes 8, 9N and 11A, should be of special vigilance in the future [19, 20, 23–25].

Serotype 24F, the most frequent serotype in IPD cases during the study period, circulated as clonal types belonging to CC230 (ST230 and the single locus variant ST4677) and was not detected in the pre-PCV13 period. Data from Spanish and French national surveillance systems identified serotype 24F as the most frequent cause of paediatric IPD in the most recent years, accounting for 11.3% (2019) of IPD cases in Spanish children <17 years and 24.4% (2015–2017) in French children <2 years [19, 25]. Interestingly, this serotype was also the most frequent NVT in these age groups and countries prior to PCV13 introduction (2.9% (2009) in Spain and 5.7% (2006–2010) in France).

Serotype 24F is not covered by PCV15 and PCV20 and has a high invasive potential, as shown in the present study as well as in two recent and much larger studies after PCV13 implementation in France and Belgium [26, 27]. Moreover, this serotype is associated with PNS and ER. Based on these findings, it is likely that vaccine and antibiotic pressure had a contributory role in the observed increases in NVT in several European countries and Canada since 2015 [19, 20, 23, 25, 28].

Serotype 11A was the second most prevalent NVT in IPD in Andalusia and NP carriage. In contrast, it ranked 15th in Spain for IPD in children aged <5 years. This serotype, covered by PCV20 but not PCV15, had a low invasive potential and was associated with ST6521, an ampicillin/amoxicillin-resistant CC156 related clone not detected in the pre-PCV13 period [24]. This variant has arisen due to genomic recombination between fragments of two main clones (11A-ST62 and NT-ST344) and the successful global clone Spain^9V^-ST156 associated with serotypes 9V and 14 [29]. ST6521 has also a greater biofilm production capacity, which may have contributed to the recent expansion of serotype 11A in acute exacerbations of chronic obstructive pulmonary disease and acute otitis media in Spain [30, 31].

The present study has several limitations. First, the study included IPD cases from three Andalusian provinces and therefore may not be fully representative of the entire region. Second, the limited study period did not allow us to analyse longer-term changes in IPD. Third, the evolution of IPD IRs in 2020 is difficult to interpret because of the potential impact of the COVID-19 pandemic on IPD epidemiology. Fourth, historical controls were collected from two hospitals that represented approximately 40% of IPD cases in the PCV13 period. Finally, the limited number of IPD and NP carriage isolates could undermine the ability to detect a statistically significant high invasive potential.

In conclusion, the proportion of IPD cases due to PCV13 serotypes declined significantly in 2018–2020 after the initiation of the PCV13 universal vaccination programme in late 2016. However, certain NVT, such as serotypes 24 and 11A, warrant future monitoring in IPD due to invasive potential and/or antibiotic resistance rates.

## Data Availability

The data that support the findings of this study are openly available in Open Science Framework at https://osf.io/profile/, reference number DOI:10.17605/OSF.IO/T4FRH.
